# Handgrip Strength, Depressive Symptoms, and Cognitive Function in Older Mexican American Adults

**DOI:** 10.1093/geroni/igaf122.3781

**Published:** 2025-12-31

**Authors:** Divya Kashyap, Soham AlSnih

**Affiliations:** University of Texas Medical Branch, Galveston, Texas, United States; The University of Texas Medical Branch at Galveston, Texas, Galveston, Texas, United States

## Abstract

High depressive symptoms (DS) are associated with cognitive decline, while high handgrip strength (HGS) is associated with lower cognitive decline. This study examined nativity differences in the relationship between HGS and DS with cognitive impairment (CI) over a 12-year period among 984 Mexican American adults aged 75 and older who scored ≥21 on the Mini-Mental State Examination (MMSE) at baseline. CI was defined as an MMSE score < 21. HGS was classified as low or high (< 27/≥27 kg for men, < 16/≥16 kg for women), and high DS was defined as a score ≥16 on the Center for Epidemiologic Studies Depression Scale. Covariates included socio-demographics and health characteristics. Participants were categorized into four groups: low HGS-high DS, low HGS-low DS, high HGS-low DS, and high HGS-high DS. Among US-born participants, those in the low HGS-low DS and high HGS-low DS groups had 59% and 60% lower odds of CI over time, respectively, compared to the low HGS-high DS group, controlling for covariates. Foreign-born participants in the low HGS-low DS, high HGS-low DS, and high HGS-high DS groups had 67%, 73%, and 80% lower odds of CI over time, respectively, compared to the low HGS-high DS group, controlling for covariates. Among foreign-born participants with high DS, high HGS was significantly associated with 24% lower odds of CI over time, but this association was not found among US-born participants with high DS, controlling for covariates. These findings suggest that interventions targeting both DS and physical strength may benefit cognitive health in this population.

